# Cases of Patients Treated in Countries With Limited Resources and Discussed by Experts of the International CML Foundation (iCMLf)—Case No. 2: Treatment-Free Remission After 9 Years of Imatinib Treatment Without Prior Achievement of Sustained Deep Molecular Response

**DOI:** 10.1155/crom/3942816

**Published:** 2025-03-21

**Authors:** Mariana Bohns Michalowski, Meinolf Suttorp, Arlene Harriss-Buchan, Guiseppe Saglio, Nicola Evans, Nirmalya Roy Moulik

**Affiliations:** ^1^Department of Pediatrics, Universidade Federal do Rio Grande do Sul, Porto Alegre, Brazil; ^2^Pediatric Hemato-Oncology, Medical Faculty, Technical University, Dresden, Germany; ^3^International CML Foundation, Bexhill-on-Sea, UK; ^4^Department of Clinical and Biological Sciences, University of Turin, Turin, Italy; ^5^Tata Memorial Hospital, Homi Bhabha National Institute, Mumbai, India

**Keywords:** DMR, imatinib, pediatric CML, stopping TKI, TFR

## Abstract

Pediatric chronic myeloid leukemia (pCML) is a rare malignancy that nowadays is treated upfront with tyrosine kinase inhibitors (TKIs). As demonstrated in adult CML patients, achieving deep molecular response (DMR) and maintaining this status over 2 years results in the opportunity to discontinue TKI therapy. Following cessation, this treatment-free remission (TFR) status is successfully achieved by approximately 50% of the patients, while the other half experience molecular relapse within ≤ 6 months, requiring a TKI restart. As pCML accounts for only 2%–3% of all childhood leukemias, experience and familiarity with this disease, especially with stopping attempts, are still very limited. Small pCML cohorts enrolled in stopping TKI trials, with strict criteria applied for both depth and maintenance of DMR, have demonstrated the achievable TFR success rates seem comparable to adults. However, recommendations for considering TFR in pCML have yet to be defined. We report on a 9-year-old Brazilian boy diagnosed with CML in a chronic phase. He was treated with imatinib and achieved a molecular response (BCR::ABL1 transcript rate < 0.1%) at Month 12. Not achieving DMR, he responded well, but not optimally, to TKI therapy. Contrary to existing guidelines on TKI cessation in adults, after 9 years, imatinib was stopped. With a follow-up of 24 months, the patient is in TFR and now maintains DMR successfully. With the support of the International CML Foundation (iCMLf), which aims to improve outcomes for CML patients globally, this rare case from Brazil is discussed from the perspective of a pediatric hemato-oncologist from a high-income country, a pediatric hemato-oncologist from a low- and middle-income country, an adult CML hematologist, and the treating physician. Sharing cases of pCML in LMICs and highlighting the resources offered by the iCMLf, particularly the Knowledge Center (available online), will hopefully improve the expertise on pCML treatment worldwide.

## 1. Introduction

Pediatric chronic myeloid leukemia (pCML) is a rare malignancy accounting for only 2%–3% of all childhood leukemias [1]. In higher income countries with greater resources, the annual incidence of pediatric chronic myeloid leukemia (CML) is approximately 100 times lower than in adults, estimated at 0.06–0.12 cases per 100,000 children per year. Incidence rates increase with age, peaking in the sixth decade of life. However, in low- and middle-income countries (LMICs), a lower median adult age and higher pediatric CML case numbers have been reported. The exact cause of which is yet to be determined [2]. Nevertheless, because of its rarity, familiarity with pCML is limited even in pediatric hemato-oncologists. Since 2001, the introduction of tyrosine kinase inhibitors (TKIs) has revolutionized the treatment of CML. In pediatric patients, as with adults, stem cell transplantation is postponed to third-line treatment, and if—after years-long therapy—a deep and sustained molecular remission is achieved, the TKI therapy may be stopped successfully in approximately half the patients [3–5]. However, evidence regarding treatment-free remission (TFR) in pCML remains limited, and formal recommendations for TFR in pCML are lacking.

The International Chronic Myeloid Leukemia Foundation (iCMLf) aims to improve the outcomes of CML globally by expanding access to world-class CML education and best practices for physicians and scientists, no matter where in the world they are located [6]. In LMICs, where resources, diagnostics, and access to medicines may be limited, this goal presents increased challenges, and healthcare teams face multiple obstacles in diagnosing and treating this rare condition in children [7]. The iCMLf addresses these problems through an extensive online, global communication network. International experts share their experience and advice with physicians and scientists through webinars, clinical preceptorships, case discussions, and educational conferences, along with tailored regional and country-specific discussion groups.

Challenging pCML cases have been presented and discussed with pediatricians during iCMLf virtual meetings across Latin America, South Asia, and Africa since 2023. Following the first publication of one such case [8], we now present the second case in our series on pCML management in LMICs. As before, key aspects of the case are discussed by a multidisciplinary team, including a pediatric hemato-oncologist from a high-income country (M.S.), a pediatric hemato-oncologist from a LMIC (N.R.M.), an adult CML specialist (G.S.), and the treating physician (M.B.M.). A.H.B. from the iCMLf provides comments from the perspective of continuing to improve the care of children with CML worldwide.

Readers from LMICs with additional interesting cases of pCML are invited to contact the corresponding author. By sharing such case presentations, we hope to promote knowledge of pCML and outline the obstacles and limitations that healthcare workers face in LMICs. Hopefully, some solutions—at least in part—are also presented.

The case described here reports on a child in Brazil diagnosed with chronic myeloid leukemia in chronic phase (CML-CP) who responded well, but not optimally, to imatinib therapy. Contrary to existing adult guidelines on TKI cessation, after 9 years of treatment, his therapy was, so far, successfully stopped without having achieved prior deep, sustained molecular response.

## 2. Case Report

In October 2013, a 9-year-old boy was diagnosed with CML-CP after presenting with nonspecific symptoms such as fatigue and mild abdominal discomfort. Physical examination revealed pale mucous membranes, absence of palpable lymphadenopathy, and notable splenomegaly (10 cm below the costal arch). These symptoms prompted further hematological evaluation.

The laboratory tests yielded the following results: white blood cell count 125 × 10^9^/L (58% neutrophils, 8% bands, 4% promyelocytes, 4% eosinophils, 6% basophils, 11% lymphocytes, 5% monocytes, and 4% blasts with myeloid morphology), hemoglobin 6.2 g/dL, hematocrit 18.6%, and platelet count 650 × 10^9^/L. Serum electrolytes, creatinine, and uric acid levels were within normal range. Serum lactate dehydrogenase was markedly elevated (> 2000 U/mL).

A bone marrow (BM) aspirate showed 12% myeloid blasts. Cytogenetic analysis and fluorescence in situ hybridization (FISH) confirmed the diagnosis of CML-CP by the presence of translocation t(9; 22)(q34; q11) in 20 out of 20 metaphases without additional abnormalities and a positive p210 BCR::ABL1 transcript. Information about the transcript subtype was not available. Cerebrospinal fluid analysis (performed at baseline before the diagnosis of CML was confirmed) showed no abnormalities.

This patient exhibited a difference in the proportion of blasts in the BM (12%) and the peripheral blood (pB) (4%). The common classification scores, such as Sokal and EUTOS, are based on the percentage of blasts in pB. Age is a major factor in the well-established Sokal score in adult patients, but it was demonstrated that this score is not justified in children [9]. The EUTOS long-term survival score (ELTS), however, was proven to identify children with poorer progression-free survival [10]. In this boy, an ELTS of 1.546 was calculated due to the presence of 4% blasts in pB, the enlarged spleen size, and the elevated platelet count [11]. Based on these parameters, the patient was defined as low-risk ELTS, just below the threshold of 1.568 separating low-risk from intermediate-risk.

Initial treatment with hyperhydration and hydroxyurea effectively managed hyperleukocytosis and preempted tumor lysis syndrome. Imatinib (Gleevec, Novartis Pharmaceuticals) therapy was initiated on day 30 after diagnosis at a dosage of 400 mg daily corresponding to 333 mg/m^2^ (patient's body surface area (BSA) was 1.2 m^2^).

The patient achieved a complete hematological response within 1 month and a complete cytogenetic response (CCyR, undetectable Philadelphia chromosome) after 6 months, as confirmed in follow-up studies. Major molecular response (MMR) (MR3 BCR::ABL1 IS = 0.1%) was attained 12 months after starting imatinib therapy. Unfortunately, limited access to the patient's medical records does not allow a more detailed analysis of the molecular kinetics of the response in the first year. Additionally, access to data from the first 4 years of treatment is hampered as results were not integrated into the electronic medical records.

**Figure 1:**
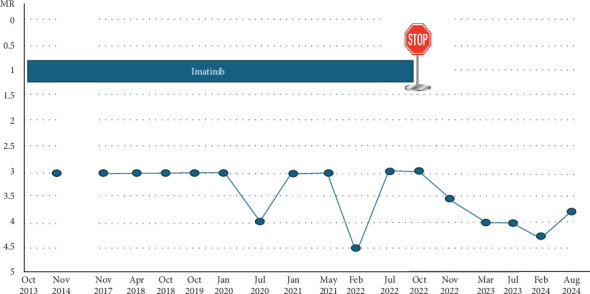
Course of molecular response (MR). The graph illustrates the patient's MR over time, measured by the BCR::ABL1 transcript levels according to the international scale. Before 2017, data were not stored electronically and are missing.

During therapy, quantitative PCR monitoring consistently showed sustained MMR with occasional fluctuations just below the MR4 threshold, but without maintaining MR4 or better for longer periods (Figure 1). The administered daily dose of 400 mg was not changed over the years despite the patient experiencing approximately 3 kg of weight loss during the seventh year of imatinib use. This was associated with loss of appetite but without gastrointestinal symptoms such as diarrhoea or vomiting.

In August 2022, the patient expressed his intent to stop the medication on his own. Additionally, he had been missing scheduled appointments after transitioning as an adult patient to the department of internal hematology. In consultation with all involved physicians, an attempt to discontinue imatinib was initiated, balancing the risk of poor adherence and uncontrolled drug intake against the patient's long-term maintenance of MMR and potential quality-of-life improvements. This decision was strongly influenced by the patient's and family's desire to discontinue medication, coupled with concerns about long-term adherence and the risk of loss to follow-up after 9 years of continuous treatment. Following discontinuation, the patient has been monitored by PCR at 3–6-months-long intervals, consistently maintaining MR3 or lower in recent assessments. Interestingly, after the cessation of imatinib therapy, an ongoing decline of the residual leukemic cell load was observed (Figure 1). Withdrawal syndrome was not reported following discontinuation of imatinib. At the last follow-up in August 2024 (24 months post-TKI cessation), he remains in deep molecular response (DMR). The small transcript rise (MR3.77) will be further investigated at the next patient visit.

## 3. Discussion

The discussion is focused on the following issues:

1. What is the optimal dose of imatinib in children?
2. What are the optimal PCR monitoring intervals?
3. Is there an optimal scenario for a TFR attempt after a decade-long TKI treatment without achieving sustained DMR? Are there other reports/experiences on this topic?
4. What could explain the observed decline of minimal residual disease without TKI intake?
5. The risks of not performing further molecular tests on this patient.
6. Role of the iCMLf in addressing the challenges outlined in this case.

### 3.1. What is the optimal dose of imatinib in children?

M.B.M.: Given the context, the boy's dose of 400 mg/day was on the higher side, aligning with dosing strategies for accelerated phase CML (CML-AP), especially at the start. However, as his BSA increased, the dose became suitable for CML-CP. Additionally, the use of one tablet of 400 mg per day was the most practical option for the patient and family, minimizing complexity and promoting adherence to therapy. In retrospect, his sustained response justified the continuation of this dose.

N.R.M.: The recommended dose for pCML-CP varies between 260 and 340 mg/m^2^ based on previous studies [5, 12–16]. We adjust doses by rounding up or down according to tablet strength (400 mg tablets and 100 mg tablets), ensuring a consistent daily dosage for patients, with the goal of delivering as close to 300 mg/m^2^ BSA as possible.

M.S.: Pediatric dosing recommendations are based on the BSA (Table 1) and are listed as a relative dose of 260–270–300 mg/m^2^ for therapy of chronic phase [12]. The maximum absolute daily dose for CML-CP is the adult 400 mg dose. Initially, this boy was treated with a single 400 mg tablet per day, which corresponds to a pretty high relative dose of 333 mg/m^2^ which is more appropriate for CML-AP. As the ELTS score was a borderline intermediate risk, this is a practicable approach [19]. As his age increased, the patient's gain in BSA automatically adjusted adaptation to the recommended dosing for CML-CP.

G.S.: In adults, the standard dose of imatinib is 400 mg/day without regard to body mass index (BMI) or BSA, but higher doses of imatinib (800 or 600 mg/day) have been tested in clinical trials and, although not always well tolerated by patients, have been reported to be associated with a deeper molecular response [27]. As reported above, the dosage of imatinib in children has been established more definitively, but this is more to avoid possible adverse events on a child's growth than based on tested outcome data. Furthermore, it must be considered that the activity of imatinib and the associated side effects are also strongly influenced by the uptake of the drug and the cellular intra- and extracellular transport systems, which have been extensively studied in adults [28], but as far as I know, never in children. Therefore, the dose used in this case, which is slightly higher than the recommended dose, is, in my opinion, fully acceptable.

Take home message: Pediatric dosing of imatinib for CML-CP is based on BSA, targeting a range of 260–340 mg/m^2^, with a maximum dose of 400 mg/day (equivalent to the adult dose). The prescribed dose should align with the available tablet strengths (400 and 100 mg), allowing for rounding up or down to the nearest tablet size. While initial dosing may be slightly higher, this approach supports adherence and naturally adjusts as the child grows.

### 3.2. What are the optimal PCR monitoring intervals?

M.B.M.: In this case, PCR monitoring occurred every 3 months but was later adjusted to longer intervals as the boy's response stabilized. This aligns with common practice to reduce monitoring frequency in patients with stable MMR.

N.R.M.: Quantitative molecular monitoring by polymerase chain reaction (qPCR) in our center costs three times that of FISH; therefore, to lower costs, we monitor by pB FISH until attaining CCyR (0% by FISH) and then switch to three monthly qPCRs until MMR (< 0.1% by qPCR). In patients with stable MMR, we increase monitoring frequency to 6–12 months if there are no concerns with compliance or fluctuations in the molecular response. Less frequent monitoring is aimed at reducing costs and accommodating patients who travel long distances to our center, allowing for longer intervals between follow-up visits. We have not encountered any significant issues in patients with stable doses and responses despite less frequent monitoring [5].

M.S.: qRT-PCR for BCR::ABL1 on pB is recommended by the I-BFM CML committee in 3-month intervals [15]. This has not been explored as a standardized approach. In the United States, the National Comprehensive Cancer Network (NCCN) recommends three monthly monitoring for the first 3 years after starting TKI therapy and then every 3–6 months [29]. The Children's Oncology Group (COG) recommends an interval of every 3 months until CCyR is achieved, then every 3 months for 2 years and thereafter every 3–6 months. If a 1-log increase is observed, but MMR is maintained, PCR should be repeated in 1–3 months [16].

G.S.: Ideally, qPCR should be performed at least every 3 months until stable MMR is achieved, and then at least twice a year. More frequently, of course, after discontinuation [30]. However, the strategy proposed above by N.R.M. is fully acceptable if resources are limited. Even in the case of discontinuation, a frequent FISH analysis on pB (every 2 months for the first year) should be completed. Indeed, in patients who persist in CCyR and are FISH negative on pB 2–3 years from diagnosis, the risk of disease progression is low. A possible molecular or even cytogenetic relapse can be addressed by switching therapy to a more appropriate TKI. It is important, however, to continue lifelong monitoring of patients not only to detect the emergence of a resistant clone (unlikely after stable MMR achievement) but also to check for adherence to therapy, which can decrease over time, especially during adolescence.

Take home message: Molecular monitoring of BCR::ABL1 in pCML should be performed every 3 months until achieving stable MMR, after which monitoring intervals can be extended to 6–12 months based on patient stability, adherence, and resource availability. While qPCR is the standard method, FISH may be used initially until CCyR is achieved to reduce costs. Guidelines vary, but the key goal is lifelong monitoring to detect potential loss in response, therapy resistance, or adherence issues, especially during adolescence.

### 3.3. Is there an optimal scenario for a TFR attempt after a decade of TKI treatment without achieving sustained DMR? Are there other reports/experiences on this topic?

M.B.M.: In this case, the decision to stop imatinib was not based on the ideal criteria for a TFR attempt but was driven by the patient's and family's desire to discontinue medication after a decade of treatment. While not typical, the patient has maintained a stable response post discontinuation, which is being monitored as closely as possible.

N.R.M.: There are no published reports of attempts to implement TFR in children who have not attained sustained DMR. In my clinic, two patients who stopped imatinib on their own accord while in MMR, but not DMR, during the COVID-19 pandemic, came back with very high transcript levels and had to be restarted on imatinib (unpublished experience).

Although when compared to adults, experience stopping TKI therapy in children is limited and characterized by a broad range of success rates (Table 2), it can be learned from this boy that a TFR journey need not align with the ideal adult criteria for cessation.

The impact of TKI discontinuation must be carefully weighed for each patient. While the desire to stop therapy is understandable—particularly when patients are stable for a long time—the risk of relapse and potential difficulty in regaining disease control if resistance develops makes it critical to approach discontinuation cautiously and within structured, evidence-based protocols wherever possible.

Adherence to treatment in children and adolescents with CML can be challenging, especially as a patient transitions into adolescence [33]. Research indicates that adherence tends to decline over time due to factors such as treatment fatigue, side effects, and the impact of long-term medication on daily life and social activities [13]. These challenges highlight the critical role of family education and support in ensuring consistent treatment.

M.S.: I am unaware of any other reports or experiences with such a case. The routine approach was outlined 10 years ago, and the available data support a minimal residual disease level of around MR4.5 to be maintained for at least 2 years [34]. However, long-term treatment, such as 9 years in this case, might also be an important factor associated with a higher probability of maintaining TFR. Other factors identified in adults include low-risk Sokal score, prior interferon treatment (neither of which apply in this patient), and higher numbers of natural killer cells at the time of imatinib discontinuation (not investigated in this patient) [35]. In a real-world cohort, the initial molecular response predicted the DMR but not the maintenance of TFR [36]. Unfortunately, data on the initial response is lacking in this patient, and in addition, this parameter is hard to compare to data from adults, as children usually are diagnosed with a higher tumor burden, causing protracted achievement of MR [8, 37].

In mathematical models of CML and TKI therapy, clinical findings confirm that the overall time of TKI treatment is a major determinant of TFR success [38]. In many patients, a lower TKI dose for the same duration of therapy is equally sufficient to the standard dose. Interestingly, the modeling also suggests that a stepwise dose reduction prior to TKI cessation can increase the success rate of TFR.

G.S.: TFR is still a largely elusive process that we know is more likely to be successful if certain disease characteristics are present at diagnosis and if a stable DMR has been achieved and maintained over a relatively long time. The deeper the molecular response and the longer the duration of TKI therapy and DMR, the higher the chances of remaining in TFR, although these chances never exceed 75%–80% even under the best circumstances [35, 39]. On the other hand, there are cases in which a stable TFR can be achieved even if therapy is stopped only in MMR, as observed in cases of pregnant women and some cases enrolled in the DESTINY trial [40]. Very occasionally, cases of spontaneous regression of a pH-positive clone have been reported [41]. So, the decision to discontinue TKI therapy in CML cannot be based only on the presence of predetermined elements such as those recommended in the ELN or NCCN guidelines—which in any case cannot give a 100% accurate prediction of success or failure—but it should be a medical decision based on other elements too, like the presence of pregnancy, toxicities, or even simply the patient's discomfort with continuing TKI therapy, as in this case [42]. On the other hand, what is the risk to the patient? Only that he may have to restart therapy at some point and perhaps reconsider stopping at a later date, still with a good chance of successful TFR at the second attempt, as recently demonstrated [43].

Take home message: TFR in pCML remains an evolving concept, with success depending on factors like the depth and duration of molecular response, overall treatment length, and patient-specific considerations. While guidelines recommend stopping therapy only after achieving sustained DMR, some cases—such as this one—suggest that TFR can still be maintained even when discontinuation occurs earlier. However, stopping TKI therapy without meeting ideal criteria carries a significant risk of relapse; therefore, it is not routinely recommended.

### 3.4. What could explain the observed decline of minimal residual disease without TKI intake?

M.S.: This is hard to comprehend. Imatinib exerts a weak immunosuppression on B-precursor cells [44]. However, limited antitumor immune effector responses are already present in newly diagnosed CML patients. This is linked to an expansion of immature myeloid-derived suppressor cells and regulatory T-cells along with aberrant expression of immune checkpoint signalling pathways [45]. The initiation of TKI therapy in CML patients is associated with immune system reactivation and the restoration of NK-cell and T-cell effector-mediated immune surveillance, though with varying frequencies depending on the levels of molecular response achieved during treatment. Therefore, after TKI cessation, the patient's immune system might have a better chance of eliminating residual leukemic cells. In a small cohort of adult patients, long-term TFR with falling levels of residual leukemic cells has been described [46]. These data are also supported by a differential equation model for CML, which classifies patients into groups according to their predicted “immunologic landscapes” [47]. While one patient group required complete CML eradication to achieve TFR, other patients were able to control residual leukemic cells after treatment cessation. A third patient group maintained TFR only if an optimal balance between leukemia abundance and immunologic activation was achieved before treatment cessation.

N.R.M.: I agree with M.S. that maintenance of TFR is a function of the immune system; therefore, I would propose that although the disease was largely well controlled with imatinib for a long time, withdrawal of imatinib released the “brakes” on the host immune system, leading to further decline in the transcript levels in this patient.

M.B.M.: The decline in residual disease after stopping TKI could be attributed to the patient's immune system playing a larger role in disease control, though this hypothesis remains speculative.

G.S.: Nobody knows. Immunological control, senescence of leukemic stem cells? In any case, this phenomenon is not exclusive to CML. It also occurs in other leukemias, such as acute myeloid leukemia, and therefore, it should be attributed to the activity of a BM control mechanism on the expansion of leukemic clones. However, the immunological or other cellular mechanisms governing this control are currently unknown.

Take home message: The ability to maintain TFR in CML may be influenced by immune system activation following TKI discontinuation. While imatinib exerts mild immunosuppressive effects, its withdrawal may allow immune surveillance mechanisms, particularly NK and T-cell responses, to better control residual leukemic cells. Some rare patients, like the one in this discussion, might experience further transcript decline postcessation, potentially due to immune-mediated clearance. However, the exact mechanisms—whether immunological control, leukemic stem cell senescence, or BM regulatory processes—remain unclear and require further study.

### 3.5. The risks of not performing further molecular tests on this patient

M.B.M.: Given the risk of late relapse, particularly in younger patients, it is important to continue regular monitoring of BCR::ABL1 levels, even though the boy has maintained molecular remission thus far [48]. While the desire to stop therapy is understandable, the risks of relapse and challenges in regaining control of the disease should be carefully considered. The role of family education and support cannot be overemphasised. Parents and patients must be educated about the risks of stopping treatment outside a controlled protocol.

N.R.M.: Although the majority of relapses postdiscontinuation occur within the first 3–6 months, relapses as late as beyond 2 years of TFR have been reported in a significant subset of patients in adult studies (about 14% of molecular recurrences were beyond 2 years of TFR) [49]. Therefore, it is important to continue monitoring transcript levels in this patient.

M.S.: Late relapses occurring in the first or second year after stopping are reported [39]. In this boy, an observation period of 24 months after stopping is encouraging, but the next monitoring results will show whether the observed half-log increase in the transcript level (from MR4.25 in February 2024 to MR3.77 in August 2024) will continue to rise or whether hopefully, it is only low-level fluctuation (Figure 1).

G.S.: If follow-up is performed at least with FISH on pB, the risk of molecular or cytogenetic recurrence may be high, but the risk of progression is low as the relapse is detected earlier. In contrast, if no follow-up is performed, the risk is the same, if not worse, than at the time of the previous diagnosis. In this case, the relapse would only be detected when there are clinical symptoms or hematological relapse, with a risk of approximately 6%–7% of cases progressing to blast crisis.

Take home message: Discontinuing TKI therapy without ongoing monitoring poses significant risks. While early relapses are more common, late relapses can occur even beyond 2 years. Without regular monitoring, relapse may go undetected until clinical symptoms or hematologic progression appear, increasing the risk of disease progression and potentially leading to a blast crisis in a small subset of patients. Inadequate monitoring can result in delayed intervention, making disease control more challenging. Educating families about these risks is essential to ensure adherence to follow-up protocols and early detection of molecular recurrence.

### 3.6. Role of the iCMLf in addressing the challenges outlined in this case—comments provided by A.H.B.

*Supporting physician/patient/family communication*. This case reflects the “real-world” dilemma of balancing the strict application of clinical guidelines while acknowledging a patient's experience and legitimate expectations, underscoring the importance of physician/patient communication and a strong therapeutic relationship. In this case, the decision to stop therapy was driven by an 18-year-old patient and his family's desire to discontinue medication after a decade of treatment, rather than physician-driven advice for a TFR attempt following ELN recommendations or NCCN guidelines [50, 51]. An important consideration is that due to the rarity of the condition and the limited data on TFR outcomes in pCML (see Table 2), recommendations for TFR in pCML have not yet been established.

The importance of educating and empowering young patients and their families with evidence-based knowledge to make informed decisions about the risks and benefits of different treatment pathways cannot be underestimated. This enables meaningful conversations with their treating physician about personalised treatment choices. In this case, the potential for withdrawal syndrome [32], the importance of regular BCR::ABL1 monitoring, and a quick restart of therapy should levels rise would have been key discussion points, alongside the wish of the patient to prevent ongoing side effects and to stop taking medication every day.

M.B.M. highlights the need for patient education, citing research that shows adherence to medication among children and young people tends to decline over time. This emphasises the importance of the physician–patient–family relationship in fostering mutual confidence, informed trust, and a shared commitment to agreed management pathways. However, there is a scarcity of high-quality, age-appropriate, and culturally appropriate educational materials, tools, and support networks for pCML. The iCMLf is developing an important new program, “Empowering Families: A pCML Education Initiative,” that seeks to meet the need for enhanced patient and caregiver education for pCML in the evolving treatment landscape. The program will provide access to information about CML, living with CML over the long term, and age-appropriate support for children and young adults with CML. There is a focus on global outreach, ensuring equitable access to relevant and relatable educational resources for pCML. This program will be available on the iCMLf website from April 2025 [6].

*Support for physicians treating pCML*. The iCMLf leadership is acutely aware of the significant challenges arising from resource limitations, including gaps in capacity, limited physician experience with pCML, a lack of expert centers, insufficient data collection and retention for analysis or follow-up, and inadequate guidance for transitioning children with CML to adult care departments.

To address these challenges, the iCMLf has developed a range of initiatives aimed at supporting physicians, improving knowledge, and enhancing collaboration in the management of pCML. These efforts focus on bridging gaps in expertise, providing access to educational resources, and fostering global connections among practitioners and experts. For physicians treating children and young people with CML, the pCML module of the iCMLf Knowledge Center [52] is a comprehensive repository on CML treatment and includes specific topics on TFR in pCML and parenting children with CML. The foundation connects practitioners treating children and young adults with CML around the world with pCML experts via an online case discussion forum [53] and through regionally focused webinars and discussion groups around the world [54].

The authors propose the development of context-specific recommendations for treating pCML, specifically addressing the challenges faced in LMICs [7]. The iCMLf, leveraging its strategic positioning and established collaborative platform for knowledge sharing, is well-placed to partner with key stakeholders to explore and coordinate the development of these recommendations, ultimately contributing to improved outcomes for children with CML.

*Access to a global network of TFR expertise*. For CML patients, there are clear benefits to achieving TFR, and this has become a treatment goal for many. As demonstrated by this case, strict adherence to defined recommendations to achieve TFR is not always possible. This is especially true in LMICs, where healthcare resources and access to treatments are limited. It can be unrealistic to follow established treatment guidelines that assume the availability of certain resources, medications, and diagnostic tools when these are unavailable [7]. This is juxtaposed against the allure of avoiding a lifetime of TKI treatment and the associated side effects and costs. Decisions around treatment discontinuation benefit from tailored and nuanced conversations between CML practitioners, their patients, and families. The rarity of CML in children compounds the challenge, as many pediatricians, primary care physicians, and even hematologists have limited experience with pCML, and they often lack access to peers with relevant expertise, particularly in resource-constrained settings. In this context, the iCMLf's global networking capabilities play a pivotal role in bridging this knowledge gap, fostering critical partnerships, and enabling the dissemination of expertise to support evidence-based, tailored care for children with CML worldwide.

## Figures and Tables

**Table 1:** Imatinib doses (median and range) administered in studies (top part of the table) or recommended (lower part of the table) for therapy of pediatric CML-CP.

**Median dose (mg/m ** ^**2**^) **(range)**	**Ref.**	**Details depicted from the article**	**Comment**
260–570 (NA)	[12]	Doses of 260 mg/m^2^ IMA provide systemic exposures similar to those of adults treated with daily doses of 400 mg.	Phase 1 pharmacokinetic study with IMA dose escalation
260 (not listed)	[17]	All 47 patients received IMA as initial treatment, with a dosage of 260 mg/m^2^/day for patients in CP.	Single-center study, comparing the OS before and after the introduction of IMA
260 (188–403)	[18]	44 patients with a median age of 11.4 years (10 months to 17 years) with newly diagnosed CML in CP.	Multicenter Phase 4 study in France
260 (not listed)	[19]	Data of 106 children were analyzed with a median age of 13.5 years (range 5–18).	Comparison of three prognostic scores in pediatric CML-CP on frontline IMA
273 (150–495)	[20]	The initial IMA dose could be analyzed in only 109/140 patients with CML-CP (*N* = 30 missing BSA data, *N* = 1 missing IMA dosage).	Retrospective, multicenter Phase 3 study in Germany
287 (161–543)	[21]	48 children with a median age at diagnosis of 9 years (range 2–15).	Retrospective study on the impact of IMA on growth in Japan
300 (220–468)	[22]	54 patients in CML-CP with a median age at diagnosis of 13.6 years (range 1.2–17.9).	A retrospective study on 54 patients from 14 centers in Poland
323 (200–347) 262 (155–352)	[23]	57 CML-CP patients, median age of 11.4 years (range 2.8–17.9), were managed in 13 Italian centers (9 pediatric and 4 adult).	Retrospective analysis. Patients managed in adult centers received a lower median dose
260–300 (NA)	[15]	260–340 mg/m^2^ orally once daily gives drug exposure similar to 400–600 mg adult dosage.	Recommendations from the International BFM Group (iBFM) Study Group Chronic Myeloid Leukemia Committee
300 (NA)	[14]	The recommended starting dose in children is 300 mg/m^2^ once daily (maximum absolute dose, 400 mg) and 400–500 mg/m^2^ for advanced-stage disease.	In this review, we discuss the optimal strategies to cure childhood CML in the era of imatinib treatment
260–340 (NA)	[24]	In pediatric CML, guidelines for therapeutic decisions have been adopted from recommendations made for adult patients. This approach seems feasible in pediatric cohorts.	Article from the *Blood* series “How I Treat”
340 (NA)	[25]	Adult doses of 400 and 600 mg equated to similar exposures in children of 260 and 340 mg/m^2^, respectively. Thus, the most commonly recommended starting dose for children is in the range of 340 mg/m^2^.	Article from the *Blood* series “How I Treat”
340 (NA)	[16]	Although some European groups recommend a lower starting dose of IMA in children with CML-CP (260–300 mg/m^2^/day), based on the results of the COG Phase II study using a higher dose of IMA that was well tolerated, our preference for the initial recommended dose of IMA is 340 mg/m^2^/day (maximum dose 600 mg).	Recommendations from the Children's Oncology Group CML Working Group
340 (NA)	[26]	33 children with solid malignancies were included in a Phase II exploratory study	A covariate model describing IMA pharmacokinetics was positively correlated with body weight and albuminemia

Abbreviations: IMA, imatinib; NA, not applicable; OS, overall survival.

**Table 2:** Published results of stopping TKI attempts in pediatric trials.

**Cohort**	**Cohort size**	**Proportion of cohort eligible for stopping**		**Proportion of eligible patients who maintained TFR**		**Proportion of the total cohort who maintained TFR**		**Ref.**
	**(*n*)**	**(*n*)**	**(%)**	**(*n*)**	**(%)**	**(*n*)**	**(%)**	
French Ped. Study IV	44	10	22%	3/10	30%	3/44	6.8%	[18]
German Study CML-Ped. II	140	7	5%	2/7	9%	2/140	1.4%	[20]
Internat. Ped. CML Study^b^	470	14	3%	4/14	28%	4/470	0.8%	[31]
Internat. Ped. CML Study^b^	495	48	9%	9/18^a^	50%	9/495	1.8%	[3]
Japan Ped. Leukemia & Lymphoma Study Group	152	22	14%	11/22	50%	11/152	7.2%	[4]
Single-center study from India^c^	186	48	25%	32/45^d^	71%	32/186	17.2%	[32]

^a^Only 18 out of the 48 patients who qualified for stopping imatinib decided to cease the TKI in a controlled stopping attempt.

^b^The criteria for molecular relapse differed between the two studies: in the first analysis published in 2019, molecular relapse after stopping was defined as loss of MR4 at any timepoint, while in the follow-up analysis published in 2021, loss of MMR was set as a threshold.

^c^The high proportion of TFR eligibility in this cohort is possibly related to the prolonged duration of TKI exposure (see full paper for further details).

^d^Of the 48 eligible patients, 45 decided to participate in the discontinuation trial.

## Data Availability

Data with the limitation that the identity of the patient cannot be disclosed are available on written request from the first author, Dr. Mariana Bohns Michalowski, Department of Pediatrics, Universidade Federal do Rio Grande do Sul, Porto Alegre, Brazil (email: mmichalowski@hcpa.edu.br).
